# The Phenomenon of Pain in Adults With Intellectual Disability: A Qualitative Systematic Review

**DOI:** 10.1111/jar.70093

**Published:** 2025-07-10

**Authors:** Alice Trainer, S. J. Summers, Alan Bowman

**Affiliations:** ^1^ Lancashire Teaching Hospitals NHS Foundation Trust Fulwood UK; ^2^ Clinical Psychology Teesside University Middlesbrough UK

**Keywords:** intellectual disability, pain, qualitative, systematic review

## Abstract

**Background:**

People with intellectual disability are vulnerable to developing and experiencing pain, indeed more pain, due to comorbidities and secondary conditions. Their pain may also be underestimated or poorly managed, due to difficulties with verbal and non‐verbal communication. Improved understanding could have positive implications for pain assessment and management practices.

**Method:**

This systematic review synthesised findings from seven qualitative studies regarding the phenomenon of pain for people with intellectual disability, using a meta‐ethnographic approach.

**Results:**

Findings offer different perspectives about the recognition of multiple causes of pain, individual differences in the expression of pain, and decision‐making about the assessment and treatment of pain. A tentative model is presented.

**Conclusion:**

There are only a small number of qualitative studies examining this topic. Further research is needed to fully understand pain for people with intellectual disability. Recognition should be given to the impact of wider factors on the pain experience.

**Summary**

- We looked at what research there is about pain for people with intellectual disability.
- We found seven studies and looked at these closely.
- We found that there are many causes of pain. There are also differences in how people express pain, and how decisions are made about assessment and treatment.
- We developed a model to try to explain this, which we hope will help with understanding and treating pain for people with intellectual disability.

## 1 Introduction

The International Association for the Study of Pain (IASP) has defined pain as ‘An unpleasant sensory and emotional experience associated with, or resembling that associated with, actual or potential tissue damage’. (International Association for the Study of Pain 2020). The implications of pain are well‐documented and there is a clear understanding that pain has widespread biopsychosocial impacts (Hadi et al. 2019; Walsh et al. 2011).

Regarding pain and people with intellectual disability, there is increasing attention being paid, but our understanding of the experience of pain within this population remains limited. Millard and De Knegt (2019), in their systematic review of cancer pain for people with intellectual disability, described the limited amount of available literature, with studies characterised by brief or unspecific descriptions of cancer pain in this population. Other reviews have examined pain in people with intellectual disability and dementia (Gabre and Sjoquist 2002); the concept of pain and how to access the experience of pain for those with the cognitive, communicative, and motor difficulties associated with intellectual disability (Symons et al. 2008); pain in adults with Down Syndrome (De Knegt and Scherder 2011); and the prevalence and assessment of chronic pain (McGuire and Kennedy 2013). However, these articles have not taken systematic approaches to reviewing the available research. These reviews highlighted that pain is largely unrecognised and untreated in people with intellectual disability, despite prevalence studies and other research suggesting an increased risk for pain‐inducing experiences (e.g., Breau et al. 2003; Nocon et al. 2008; Robertson et al. 2017).

El‐Tallawy et al.'s (2023) narrative review of pain management for people with an intellectual disability explored the prevalence, risk factors, causes, diagnosis and possible management of pain; pain assessment issues; and practical guidance. They concluded that this population is amongst the most vulnerable to developing and experiencing pain, and indeed more pain, due to comorbidities. In addition, they found that people with intellectual disability are more likely to have underestimated or poorly managed pain, due to difficulties with verbal and non‐verbal communication of the pain experience. It was argued that effective methods for pain assessment and treatment are a challenge, with a focus needed on involving people with intellectual disability and their caregivers in the assessment and management of pain. The authors proposed that training for parents and carers should aim to increase knowledge and awareness of how to manage pain experiences for people with intellectual disability. Promising evidence was found regarding the incorporation of neuroimaging and electrophysiological methods in pain assessment, and for new technologies such as virtual reality for helping with pain management. Further research was recommended to learn more about risk factors, prevention, assessment, and management of pain for people with intellectual disability.

Goodall et al.'s (2023) mixed methods systematic review aimed to synthesise research regarding the recognition, assessment, and perception of pain in people with profound and multiple intellectual disabilities, with the aim of understanding how pain is understood and conceptualised in this population. This highlighted difficulties in pain recognition, assessment, and perceptions of pain. The main challenge was the difficulty in understanding and giving voice to this group, who have unique and individual means of communication. The authors argued that the knowledge held by caregivers is not shared in research, which would facilitate a deeper understanding of how to help people with profound intellectual disability with their pain.

A fuller understanding of the experience of pain for people with intellectual disability and those who support or care for them could have positive implications for pain assessment and management practices. This review therefore sought to systematically examine qualitative studies examining the phenomenon of pain for adults with intellectual disability. Specifically, the following question was asked: What does the published qualitative literature tell us about the phenomenon of pain within the adult intellectual disability population?

The ‘phenomenon of pain’ was inclusive of both the embodied experience of pain and its impact, including experiences of assessment and treatment of pain.

## 2 Method

### 2.1 Search

This review was conducted in line with the Preferred Reporting Items for Systematic Review and Meta‐Analyses (PRISMA) guidelines (Page et al. 2021).

Eight electronic databases: PsycInfo; AMED; Medline; PsycArticles; CINAHL Complete; ASSIA; EBSCO; and the Psychology and Behavioural Sciences Collection. These were searched in two stages: in February 2022 and then in July 2024—to establish whether any further studies had been published since the first stage. Reference lists of accepted articles were also hand screened.

Search terms were identified and refined through scoping searches of the selected databases using keywords on the topics under study. These were kept deliberately broad, as the review was focused on the experience, recognition, assessment, and management of pain by adults with intellectual disability, and/or their parents or carers, and/or health and social care professionals. Boolean operators were used to maximise results where databases allowed for this; otherwise, individual search terms were used per search line. Specific search terms related to research design were excluded in order to maximise opportunities to identify all qualitative pieces of research which may have been coded ambiguously and otherwise excluded in search results. See Table 1.

**TABLE 1:** Summary of search terms.

		Search terms
Population	Adults with intellectual disability (18 or over); health and social care professionals working with or coming into contact with adults with intellectual disability; and parents or carers of adults with intellectual disability	‘Learning disability’ OR ‘learning disabilities’ OR ‘intellectual disability’ OR ‘intellectual disabilities’ OR ‘developmental disability’ OR ‘developmental disabilities’
Phenomenon	Pain	‘Pain’

Inclusion and exclusion criteria are presented in Table 2.

**TABLE 2:** Inclusion and exclusion criteria.

Inclusion criteria	Exclusion criteria	Rationale
Population		
People with intellectual disability; or health and social care professionals working with or coming into contact with people with intellectual disability; or parents or carers of people with intellectual disability	People without intellectual disability; or health and social care professionals not working with people with intellectual disability; or parents or carers of people without intellectual disability	To answer the review question, only studies that included either people with intellectual disability, their parents or carers, or health and social care professionals working with this population are included. Any level of intellectual disability is included. Any level of experience working with or caring for people with intellectual disability is included.
Adults (over the age of 18 years old)	Children (under the age of 18 years old)	To consider the evidence in relation to the current study investigating the phenomenon of pain within the adult intellectual disability population.
Domain under study		
Studies examining the phenomenon of pain	Studies examining phenomenon other than pain	To answer the review question, only studies that aimed to examine the phenomenon of pain in this population were reviewed. Studies examining other phenomenon, where the exploration of pain was not specifically included were excluded from this review to ensure that pain remained the topic under specific study.
Study characteristics		
Articles written in English and accessible before 1 July 2024	Full‐text not available	Pragmatic reasons
Published in a peer‐reviewed journal	Non‐peer reviewed journal articles (e.g., commentaries, clinical recommendations, thesis, newspaper articles)	To try to ensure quality of studies included in the review
Qualitative study design or qualitative phase of mixed methods studies where the qualitative aspect is reported separately with clear division between qualitative and quantitative data	Quantitative study design or mixed methods studies where the qualitative aspect is not reported separately and without clear division between qualitative and quantitative data or case reports presented in a purely descriptive manner excluding any data analysis	To answer the review question, only qualitative study designs or distinct qualitative aspects of mixed methods studies are included in the review.
Any publication date		To ensure all relevant studies are included in the review

### 2.2 Quality Appraisal

Critical Appraisal Skills Programme (2018) checklist for qualitative studies was used to appraise quality. Items are graded as ‘yes’, ‘no’, or ‘can't tell’. CASP recommends using the checklist as a guiding tool in assessing the quality of relevant research. The checklist may not produce results that can be used for weighting or organising studies based on quality, and objectivity in pursuing this is difficult in the appraisal of qualitative research (Long et al. 2020). Therefore, articles were not assigned a total quality rating score, and quality appraisal served a descriptive function in this review. No studies were excluded from the review following quality appraisal.

### 2.3 Triangulation

For the first stage of selection, the first and second authors independently assessed full‐text articles against the criteria presented in Table 2. This process was repeated for the second stage by the second and third authors. For both stages, there was full agreement regarding the studies to be included, and reasons given for inclusion and exclusion were the same for both reviewers.

In relation to quality, the first and second author assessed all included articles, and the third author assessed a random sample of three included articles. Initially there was a fair level of agreement (77.8%; *k* = 0.36) regarding quality grades. Discussions outlined reasons for differences in quality appraisal scores and, following this, agreement on final quality appraisal scores was reached.

### 2.4 Data Extraction

A data extraction tool was developed to capture and organise key aspects of the included studies. See Table 3.

**TABLE 3:** Key characteristics and findings of included articles.

Author/s (Year)	Phenomena of interest/aim	Methodology (type, theory/framework)	Methods (sampling, data collection and data analysis)	Setting	Participants (number, sample and age)	Findings (key outcomes and conclusions)
Clarke and Thompson (2007)	Parents' experience of pain in their minimally or non‐verbal adult child; recognition and response of parent	Qualitative; IPA (Smith et al. 2009)	Purposive sampling; individual semi‐structured interviews; audio‐taping, transcription, reading and re‐reading, note‐making, noting themes (emerging, dominant, master)	Health respite service	*N* = 8 parents, age range 44–84	Parents did not often perceive son or daughter to be in pain; parents had specific ways of recognising pain and many used a trial and error approach to determine cause; parents develop strategies for dealing with pain and have mixed experiences of involving services
Chester and Henriksen (2014)	Experience and management of pain with people with intellectual disability in a forensic setting	Mixed methods; baseline audit, descriptive qualitative component	Purposive sampling; semi‐structured interviews, Health Action Plans (HAPs) and medication files; common themes and language presented	Inpatient forensic intellectual disability service	*N* = 64 patients, no age range; *N* = 12 nurses, no age range	Different causes of pain described including restraint, assault and self‐harm; awareness of impact of pain on daily living, emotions and mental state however lesser understanding of impact of emotions on physical symptoms; individuality in whether pain is reported influenced by health myths and other factors; nurse decision making affected by individual beliefs.
Donovan (2002)	Experiences of intellectual disability nurses when working with minimally or non‐verbal people with intellectual disability who may be in pain	Qualitative; phenomenology (Hycner, 1985)	Snowball sampling; unstructured interviews; audio‐taping, transcription, bracketing suppositions, focusing on key themes and seeking the opinion of professional colleagues	Residential care homes	*N* = 8 intellectual disability nurses, no age range	Five central themes: the importance of a caring relationship with the client; recognising changes in verbal and non‐verbal behaviour; searching for a meaning in the client's behaviour; negotiating with other health professionals; and sharing in the client's feelings. Wide range of both conventional and nonconventional forms of nonverbal communication used to express pain.
Drozd et al. (2020)	Experience of people with intellectual disability of trauma and orthopaedic hospital care	Qualitative; IPA (Smith et al. 2009) cross‐case analysis	Purposive sampling; 1:1 semi‐structured interviews; audio‐recording, transcription, accounts and themes analysed independently by three authors, individual superordinate and subordinate themes cross‐compared and master themes developed	Self‐advocacy groups; national organisations for PwLD; healthcare professional members; service user and carer group	*N* = 4 PwLD, age range 25–45; *N* = 1 parent carer, no age recorded	Common and interconnected experiences across participants: communication challenges; lack of person‐centred care; issues related to pain management; lack of confidence in hospital care; valuable support and expertise of carers; incompetence of hospital staff; and isolation and loneliness.
Drozd et al. (2021)	Experience of people with intellectual disability of trauma and orthopaedic hospital care	Qualitative; IPA (Smith et al. 2009) idiographic analysis of accounts	Purposive sampling; 1:1 semi‐structured interviews; audio‐recording, transcription, exploratory observational and reflective notes, emergent patterns and themes identified	Self‐advocacy groups; national organisations for people with intellectual disability; healthcare professional members; service user and carer group	*N* = 4 PwLD, age range 25–45; *N* = 1 parent carer, no age recorded	An overall lack of person‐centred care impacted on communication challenges which in turn impacted on assessment and management of pain; issues of isolation and loneliness negatively impacted on trust in the hospital. System; reliance on carers for support while in hospital; all participants experienced unmanaged pain while in hospital despite most being able to verbally report pain.
Findlay et al. (2014)	Experience and understanding of pain	Qualitative; content analysis (Elo and Kyngäs 2008)	Purposive sampling; 1:1 semi‐structured interviews; audio‐taping and transcription, developing categorisation matrix, coding data, grouping codes to categories and abstracting subcategories	Specialist health team for adults with intellectual disability	*N* = 15 people with intellectual disability, age range 21–61	Pain was described using negative meanings and strong imagery; various causes of pain were given; minimal description of pain management or coping with pain; differences in the reporting of pain to others with some choosing to hide these experiences; general belief that others can tell when someone is in pain. Possessing verbal skills cannot be taken as an indicator that pain will be communicated.
Findlay et al. (2015)	Experience of caregivers supporting people with intellectual disability experiencing pain	Qualitative; IPA (Smith et al. 2009)	Purposive sampling; 1:1 semi‐structured interviews; audio‐taping, transcription, coding, clustering into themes, identifying shared themes, developing superordinate themes	Carers group; residential care homes	*N* = 5 formal caregivers, *N* = 6 parent caregivers, age range 29–67	Six superordinate themes: suffering in silence; searching for meaning to explain the complaint; knowledge and skills needed to recognise and manage pain; acting to try and reduce pain; the emotional impact of pain. Recognising and treating pain was experienced as complex and ambiguous. Some carers described a negative emotional impact of pain and dissatisfaction with pain management by health services.

### 2.5 Data Synthesis

Data were synthesised using a meta‐ethnographic approach (Noblit and Hare 1988); a theory‐based interpretive methodology for qualitative synthesis widely used in healthcare research (Campbell et al. 2011). Meta‐ethnography involves seven stages: (1) determining the appropriateness of an aggregative synthesis; (2) deciding what is relevant; (3) reading included studies and presenting study characteristics; (4) determining how studies are related; (5) translating studies into one another; (6) synthesising translations; and (7) expressing the synthesis (Toye et al. 2014). Regarding phases 4 and 5, each study was read in its entirety before focusing on the findings and outcomes of each. A note was made of key themes and concepts drawn out of the analysis by the original researcher(s). Once this process was complete for each individual study, a constant comparative method was used (Noblit and Hare 1988) to detail reciprocal and refutational translations and, where available, any appropriate contextual information alongside these. Reciprocal translation relates to instances of agreement in meaning across studies, and refutational translation relates to instances of disagreement or contrast in the interpretations of similar concepts across studies (Toye et al. 2014). The aim was to specifically look at how the studies related to each other and the review question. Where alternative interpretations or explanations were considered by the reviewer, these were described.

Once key themes and concepts had been extracted and comparisons made, phase 6 involved data synthesis. Within this process the synthesised data represented the interpretations made by the original researcher(s), and further interpretations made by the reviewer. Translations were synthesised through the development of overarching concepts or descriptors which were deemed to adequately capture the experiences or processes being interpreted within the data across the studies.

The first author conducted the analysis, with each step discussed with the other authors. The first author recognised within this that a process of ‘categorisation’ may not align with her own beliefs and understandings about how language is used by individuals, and remained aware of how her own biases or beliefs were impacting on the review process. Where ambiguous or unique language was reported in the original study, this was included to remain as grounded in the original data and context as possible, whist seeking to appropriately synthesise the available data in relation to the aims of the review.

A researcher's ontological and epistemological position affects research questions, designs and analyses (Yeganeh et al. 2004). The first author considered her own position, recognising that her perception is that within the literature there is evidence of ‘multiple realities’: those of the participants; those of the original researchers analysing the data; and those of the reviewer herself in synthesising and interpreting the data. In efforts to ensure transparency throughout the research process, she kept a reflexive logbook to examine her own beliefs, experiences, and understandings, and consider where these may be interacting with analytic or interpretative choices in the review process. This was discussed regularly with the other authors (Olmos‐Vega et al. 2022).

## 3 Results

**FIGURE 1:**
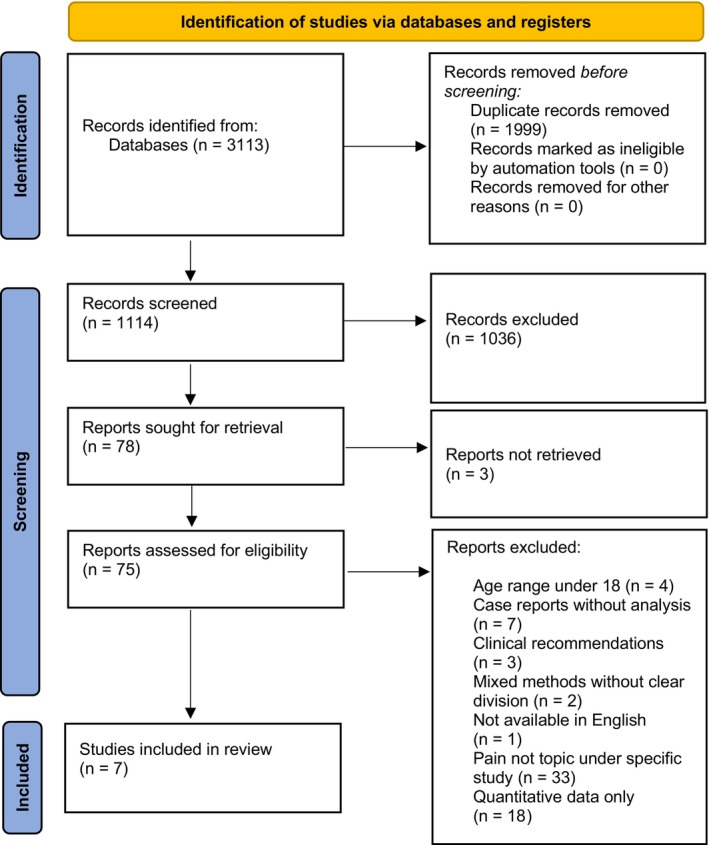
PRISMA flow diagram (Page et al. 2021).

Three thousand one hundred thirteen results were identified across all databases, of which 1999 duplicates were identified and removed before screening. In the first stage, 818 titles and abstracts were screened against the criteria outlined in Table 2. Following this, 78 articles were sought for retrieval. Of these, three articles were not available, resulting in 75 studies included in the full text review. In the second stage, 296 titles and abstracts were screened, resulting in 10 sought for retrieval; all articles were available. Finally, seven articles met the inclusion criteria and were included in the review. The second stage resulted in no new papers being identified for inclusion. Hand screening resulted in no further articles being identified. See Figure 1.

### 3.1 Study Characteristics

There were 83 people with intellectual disability (aged 18 and over); 15 parents or primary caregivers; and 25 nurses, keyworkers, or paid formal carers across the included studies. Although people with intellectual disability were more represented than carers or health professionals, 64 of these participants were involved in a qualitative component of a mixed methods audit study. The design of the studies was either qualitative (*n* = 6; Clarke and Thompson 2007; Donavan 2002; Drozd et al. 2020, 2021; Findlay et al. 2014, 2015) or mixed methods with a distinct qualitative component (*n* = 1; Chester and Henriksen 2014). The methodology of qualitative studies was interpretative phenomenological analysis (IPA; *n* = 4; Clarke and Thompson 2007; Drozd et al. 2020, 2021; Findlay et al. 2015), phenomenology (*n* = 1; Donovan 2002), and content analysis (*n* = 1; Findlay et al. 2014). Studies were typically conducted within community health services, community groups and organisations, or residential or inpatient health services for people with intellectual disability. All studies were conducted within the UK.

### 3.2 Quality Appraisal

Using the CASP checklist for qualitative studies (Critical Appraisal Skills Programme 2018), as detailed in 2.2, aspects of higher quality within all studies included: (a) clear statements of aims, goals, and relevance of the research; (b) appropriateness of using qualitative methodology; (c) justifiable research designs appropriate for the aim of the study; and (d) clear and explicit statements of findings discussed in relation to the original research question.

Limitations included: (a) a lack of critical examination of the researcher's own role, bias and influence across the research process including data collection and analysis; (b) insufficiently detailed and in‐depth descriptions of the data analysis process; and (c) insufficiently detailed consideration for, and management of, potential ethical issues. Across most studies, reflexive practice, processes for data analysis, and ethical considerations were mentioned. However, these were often presented with little in‐depth description. For example, studies mentioned the use of reflexive diaries and bracketing, but researchers did not state their own epistemological stance. See Table 4.

**TABLE 4:** Quality appraisal.

	1	2	3	4	5	6	7	8	9	10 (Comments regarding value of study)
Qualitative study checklist (Critical Appraisal Skills Programme 2018)										
Clarke and Thompson (2007)	Y	Y	Y	Y	Y	Y	Y	Y	Y	Findings discussed in the context of previous research; further ideas for research identified and suggested; practical implications discussed.
Chester and Henriksen (2014)	Y	Y	Y	Y	Y	N	Y	N	Y	Detailed explanation of policy and practice changes made in service as a result of study; exploration of how findings can be taken forward in a broader context; no further research ideas discussed.
Donovan (2002)	Y	Y	Y	Y	Y	Y	Y	Y	Y	Findings discussed in the context of previous research; practice implications discussed; potential widening of the scope and context of the findings discussed.
Drozd et al. (2020)	Y	Y	Y	Y	Y	C/T	Y	Y	Y	Unique contribution to literature; ethical, legal and professional issues raised in findings discussed; findings placed in the context of a broader body of literature; suggestions for practice changes, training and education; contribution of people with intellectual disability within research discussed.
Drozd et al. (2021)	Y	Y	Y	Y	Y	C/T	C/T	Y	Y	Mapping of themes onto Royal College of Nursing criteria; suggestions for changes to practice; areas of interest for research suggested; suggestions made for ongoing co‐production efforts with people with intellectual disability, intellectual disability nurses and experts by experience.
Findlay et al. (2014)	Y	Y	Y	Y	Y	C/T	Y	N	Y	Comparison and discussion of findings in the context of previous literature; practical implications outlined; future research directions discussed at length.
Findlay et al. (2015)	Y	Y	Y	Y	Y	C/T	Y	Y	Y	Findings discussed in the context of previous literature, practice tools and law/policy; recommendations and considerations for practice discussed; future research directions suggested.

Abbreviations: C/T, can't tell; N, No; Y, Yes.

### 3.3 Data Translation and Synthesis

#### 3.3.1 Summary of Findings

There were clear similarities in the concepts described across all the included studies. These concepts included the causes and experiences of pain, pain expression, pain recognition, pain assessment, and pain management for people with intellectual disability.

#### 3.3.2 Causes and Experiences of Pain

Whilst the reporting of the cause or location of pain experienced by participants across studies was not consistent, each study described, in varying detail, at least some of the reasons why people with intellectual disability were experiencing pain. Common causes and locations of pain included fractures; injuries; hip, back and knee pain; toothache; ulcers and hernias; abdominal pain; musculoskeletal problems; migraines; and menstrual pain (Clarke and Thompson 2007; Chester and Henriksen 2014; Donovan 2002; Drozd et al. 2020, 2021; Findlay et al. 2014, 2015). Alternative causes for pain were captured within a forensic setting, and these included physical restraint, assault, and self‐harm (Chester and Henriksen 2014). Additionally, there was pain related to medical procedures and operations (Donovan 2002; Drozd et al. 2020, 2021).

The recognition of the biopsychosocial impact of pain was evident. People with intellectual disability recognised the impact of pain on their physical wellbeing, including mobility and sleep disturbances; their enjoyment and participation in daily activities; and their overall quality of life (Findlay et al. 2014). The impact on daily activities and quality of life was also a concept noticed by parents and carers (Chester and Henriksen 2014; Findlay et al. 2015). The emotional impact of either experiencing pain as a person with intellectual disability, or caring for someone in pain, was a key concept across studies. People with intellectual disability described feeling low, angry, and helpless when experiencing pain; worried and upset about the reactions of others; disbelieved and disappointed when pain was not acknowledged or managed; confused and suspicious when communicating with health services; a lack of control when in hospital or under medical care; and untrusting of medical staff, particularly when given conflicting information and advice (Drozd et al. 2020, 2021; Findlay et al. 2014). Parents and formal carers used terms such as ‘worry’, ‘concern’, ‘distress’, ‘anger’, and ‘frustration’ when the person they cared for was in pain. Some described feeling stressed, frightened, and helpless, and dealing with uncertainty about the pain being experienced. Empathy was a common theme, with some parents experiencing ‘vicarious’ pain and it being ‘painful’ to see someone in pain (Findlay et al. 2015, 116).

Frustrations were noted in interactions with health services, alongside worries and fears about, for example, loss and dying, or about the future. Themes of guilt and self‐directed anger were also common, particularly in instances where pain had been recognised after some delay (Clarke and Thompson 2007; Findlay et al. 2015). There was much less recognition and discussion about the emotional impact of being with, and caring for, people with intellectual disability in pain within the studies with nursing staff. However, the concept of ‘sharing in feelings’ with people with intellectual disability was raised by intellectual disability nurses as something which facilitates supportive understanding and management of pain (Donovan 2002).

#### 3.3.3 Pain Expression

All studies included descriptions and consideration of how pain is expressed by people with intellectual disability. Pain was expressed in various ways via more and less conventional means, using both verbal and non‐verbal communication. Nurses described people with intellectual disability expressing pain through non‐verbal cues such as crying, increased non‐verbal sounds, and changes in mood and behaviour (Donovan 2002; Chester and Henriksen 2014). Similarly, parents and formal carers described people with intellectual disability expressing pain through verbal and non‐verbal means and highlighted that the expression of pain is highly individual (Clarke and Thompson 2007; Findlay et al. 2015). People with intellectual disability described expressing pain through spoken language, using more conventional pain language such as ‘painful’ or ‘ache’, or less conventional language such as ‘trouble’ (Drozd et al. 2020, 2021; Findlay et al. 2014). In one study, analysed in two parts, people with intellectual disability described shouting, screaming, and crying (Drozd et al. 2020, 2021), however, verbal expressions of pain without the use of language were not described elsewhere by people with intellectual disability. In another study, the use of strong language and rich imagery to describe the severity of pain was reported, for example pain is ‘murder’ (Findlay et al. 2014, 362).

Across studies, both people with intellectual disability and parents or formal carers described pain being deliberately hidden, or communication about pain being withheld or not reported. People with intellectual disability described sometimes choosing to hide pain for fear of reactions by others, for example worrying a health professional might ‘snap’; not wanting to waste others' time; not seeing clear benefits in telling others; and not being aware they could tell others (Drozd et al. 2020, 2021; Findlay et al. 2014). Parents, formal carers, and nurses proposed that possible influencing factors also included fear of visiting a doctor; fear of going through treatment procedures; and previous negative experiences, for example pain being ignored (Clarke and Thompson 2007; Donovan 2002; Findlay et al. 2015).

#### 3.3.4 Pain Recognition

Concepts relating to how pain is recognised by others were included in the outcomes of all studies. Parents, formal carers, and nurses all described difficulties in recognising when the person they were caring for was in pain. There were some common concepts expressed about needing to remain observant to subtle changes in an individual's usual presentation, and that this was reliant on the carer or health professional having a well‐developed understanding of, and relationship with, the person they were caring for (Findlay et al. 2015; Donovan 2002). In contrast, nurses in one study expressed an assumption that if an individual communicates verbally and functions well that this would mean they would verbally communicate their pain and ask for help (Chester and Henriksen 2014). People with intellectual disability described varying experiences and beliefs about whether others would recognise pain, with some expressing that others do, and some expressing that others do not, notice (Findlay et al. 2014).

Beliefs about pain and issues of diagnostic overshadowing (pain being attributed to the intellectual disability or co‐occurring mental health difficulty rather than investigating alternative causes) appeared to impact the likelihood that pain would be recognised or considered as a reason for changes in behaviour or functioning. For example, some nurses working in a forensic inpatient ward described the focus being on mental health rather than physical health and that pain was less commonly thought about (Chester and Henriksen 2014). Parents and formal carers described some beliefs about people with intellectual disability finding pain more tolerable and not feeling pain in the same way as people without intellectual disability (Clarke and Thompson 2007; Findlay et al. 2015). There was also a questioning about the genuineness of expressions of pain (Chester and Henriksen 2014; Clarke and Thompson 2007; Findlay et al. 2015). The findings of one study differed in relation to this, with intellectual disability nurses describing working from an assumption that an individual will be experiencing pain if there is a known painful stimulus (e.g., a painful medical or investigative procedure), whether or not expressions of pain are observed (Donovan 2002).

#### 3.3.5 Pain Assessment

There was varying detail regarding pain assessment in studies. There was limited discussion about pain assessment by people with intellectual disability, outside of recalling that carers ‘asked questions’ (Findlay et al. 2014, 362), or not being regularly asked about pain (Drozd et al. 2020, 2021). A shared concept amongst parent responses was in describing the difficulty with assessing pain. This was in relation to determining the severity of pain being experienced, and accurately determining the cause and location. Some parents described using guesswork, acting as being a ‘detective’ (Findlay et al. 2015, 115) and putting careful thought and effort into assessing any pain being communicated (Clarke and Thompson 2007; Findlay et al. 2015). Given potential difficulties around the communication of pain, and potential barriers and beliefs people with intellectual disability may hold about sharing pain experiences with others, some parents described asking directly about pain. However, it was noted by formal carers that in some settings this may feel inappropriate or ‘not socially acceptable’, although they acknowledged it was likely useful (Findlay et al. 2015, 115).

Nurses working in a forensic setting gave limited detail about pain assessment processes, other than describing asking about pain location and duration (Chester and Henriksen 2014). Conversely, intellectual disability nurses shared various processes, including interpreting the meaning of non‐verbal communication such as body positioning; assessing physiological changes and sensitivity to touch; assessing changes from baseline or usual presentation; and the use of body scales and maps (Donovan 2002). They also highlighted the importance of conversation and action within pain assessment, noting that there are often delays in accessing objective investigations to determine possible causes of pain, such as x‐rays or blood tests. A concept within the responses of intellectual disability nurses, which was not captured elsewhere, was a recognition that observable functional and language abilities are not a good representation of whether someone is experiencing pain or will communicate about this. Some intellectual disability nurses theorised that this could be a management strategy used by some individuals when in pain, in that not altering daily routines and activities itself may be a comfort.

#### 3.3.6 Pain Management

Pain management was discussed in all studies and many similarities were evident. People with intellectual disability, parents, formal carers, and nurses spoke about the use of pain medication; approaching health care staff and services for support; and increasing comfort levels, for example, through the use of hot water bottles, baths, and relaxation (Clarke and Thompson 2007; Chester and Henriksen 2014; Donovan 2002; Drozd et al. 2020, 2021; Findlay et al. 2014, 2015). Specific investigations and treatments, for example, surgery, were described by people with intellectual disability and nursing staff, as well as the consideration of non‐medical management such as increasing the amount of sleep and rest, and increasing fluid intake (Donovan 2002; Drozd et al. 2020, 2021; Findlay et al. 2014).

The importance of support from carers and family was highlighted as part of pain management processes by both people with intellectual disability and parents or formal carers. The importance of carers being included in care and treatment as trusted, caring, knowledgeable others was described, and some people with intellectual disability expressed this as important when in pain. Support, advice and reassurance from family and carers was described as helpful, both in terms of a calming presence impacting on emotional responses to pain, having a trusted other there for advice or to take action, and in the context of connection to others while in hospital (Donovan 2002; Drozd et al. 2020, 2021; Findlay et al. 2014, 2015).

There were varying descriptions of how pain management is approached. Parents and formal carers described processes such as ‘trial and error’ (Findlay et al. 2015); and monitoring and noticing changes (Clarke and Thompson 2007). Nurses in a forensic setting more frequently described responding to acute pain experiences, for example providing analgesic medication, without descriptions of follow‐up or monitoring. Repeated offers of pain medication were not evident from the descriptions, with nurses rather describing waiting for individuals to communicate that this was needed (Chester and Henriksen 2014).

People with intellectual disability reported the limited effectiveness of pain medication. For example, most participants in Findlay et al.'s (2014) study spoke about medication being ‘of little or no help’ (p. 362) or feeling that different or stronger pain medications may sometimes be appropriate and helpful. Delays between communicating about pain or asking for support, and pain medication being received, were also noted. Intellectual disability nurses theorised that there was a learning process for people with intellectual disability in self‐managing pain and how this could be supported by staff, for example, noticing that pain from an injury reduced with rest and thus encouraging the use of this strategy in future pain experiences (Donovan 2002).

### 3.4 Further Conceptual Insights and Model

**FIGURE 2:**
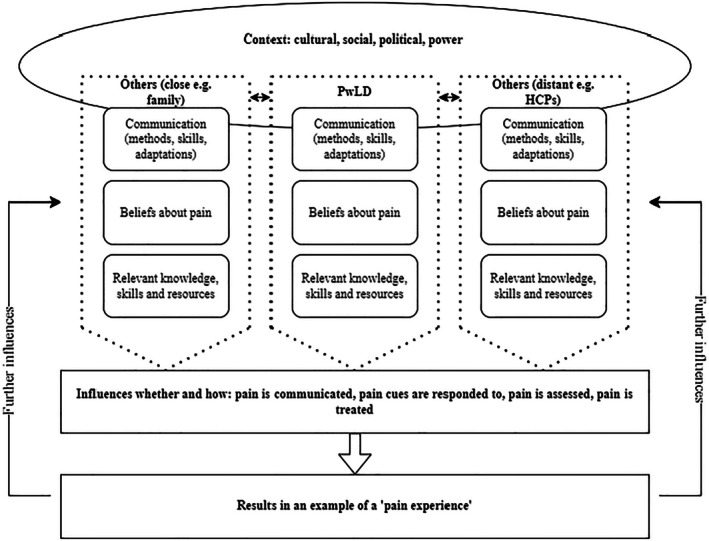
Conceptual model.

The synthesis offered some understandings about the phenomenon of pain, including insights into how pain is experienced and managed by people with intellectual disability and those who care for them. In line with the meta‐ethnographic approach (Noblit and Hare 1988), the first author additionally sought to develop and offer some further conceptual insights from this synthesis. It is important to note that these represent exploratory reflections, given the relatively small amount of qualitative research available. The model reflects the impact and interaction of both internal and external factors, in context, that influence the experience of pain for people with intellectual disability. See Figure 2.

Overarching themes were noted which ran through the concepts of pain expression, recognition, assessment, and management. Individual differences related to beliefs about pain, skills and knowledge, and communication challenges and efforts stood out as factors which interacted with pain‐related decisions and actions throughout the process. It appeared that these factors impacted, directly or indirectly, on decisions made about the expression, assessment, and management of pain. This process, taken as a whole, contributes to a learning process and becomes an example of an experience of being in pain for people with intellectual disability. This then may act as a type of feedback loop which further informs people with intellectual disability's beliefs, knowledge, and communication efforts in the event of further pain experiences.

This process is conceptualised as ongoing for both the individual experiencing pain and for those who may be supporting them. For each person involved, their own decision‐making and subsequent actions are filtered through their own understandings, experiences, skills, and resources. These may be similar or different between the individuals involved, and this may contribute further to the context within which the experience takes place. There appeared to be differences, for example, in the beliefs of intellectual disability nurses as opposed to nurses working within a forensic setting. Within the model, these beliefs are conceptualised as being individual to the nurse and are also seen in their interaction with the beliefs of the person for whom they are caring. These beliefs, in conjunction with challenges and modes of communication, and existing skills and resources, then filter the approach of the nurse and the actions taken. This is occurring at the same time as a similar filtration process for people with intellectual disability and for parents, carers, or others involved. This broad process, the actions taken, and the resulting outcome then represent a single experience of pain. The feedback from this experience then further informs the beliefs, knowledge, and skills of each person involved and contributes further to their own subjective understanding of pain.

It is likely that other overarching factors, such as the cultural context within which the person with intellectual disability lives, will influence their experiences of pain and interactions with others regarding this. The political and social climate impact directly on healthcare provision and priorities, which may influence the availability of resources, and the likelihood of particular actions being taken. Where healthcare services are involved, there are decisions and actions which are reliant on certain individuals. For example, in order to request objective investigations or prescribe pain relief, a health professional may be required. In this context, health professionals therefore hold inherent power about the actions that can be taken, and therefore how that individual pain event is experienced. This may be an issue in cases where pain has been communicated, or managed, ineffectively, given that one of the apparent themes in the existing data is about pain communication sometimes being withheld or hidden. In these instances, prior experiences could contribute to amendments in the beliefs about pain (e.g., ‘there is nothing anyone can do about my pain’); whilst also reflecting the context of imbalanced power between individuals which may further impact on pain beliefs (e.g., ‘I am powerless when in pain’). In this way, these dynamics and contextual factors may also influence the weighting of the three input factors at the top of the model (communication; beliefs about pain; and knowledge, skills and resources), but have not been explicitly drawn out of the existing data.

The model is intended to depict both the internal and external factors impacting on how pain is experienced by people with intellectual disability; through which beliefs, understandings, skills, and knowledge filter how pain is approached and therefore how pain is conceptualised and experienced. Within the data captured in existing literature, there are indications of factors which may result in pain being experienced in more and less helpful and supportive ways. These factors interact both within individuals and between them, particularly for individuals who may be reliant on others to, for example, access services on their behalf. Within the model there are opportunities for this process to result in learning both for people with intellectual disability and those in positions of care for people with intellectual disability. This highlights the importance of developing a better understanding both about the beliefs that are held with regard to pain and people with intellectual disability, and factors that support effective communication about, and management of, pain for this population.

## 4 Discussion

This review aimed to establish the current qualitative evidence base examining the phenomenon of pain for people with intellectual disability, and to provide conceptual understandings from the synthesis of this data. This aim was achieved through the application of a meta‐ethnographic approach which included the development of a conceptual model.

### 4.1 Clinical and Research Implications

The findings and synthesis provide some insight into the phenomenon of pain for people with intellectual disability. The data synthesised was collected in studies with people with intellectual disability (aged 18 or over), parents, formal carers, nurses working in forensic settings, and intellectual disability nurses. The findings contribute some understandings from these different perspectives about the recognition of multiple causes of pain, individual differences in the expression of pain, and decision‐making about the assessment and treatment of pain. The conceptual model indicates that the decisions made about expressing, assessing, and treating pain are influenced by a multitude of factors, including beliefs about pain and relevant skills, knowledge, and resources. These factors interact both within and between individuals involved (people with intellectual disability, parents, carers, and healthcare professionals) and act as a filter to the process of decision‐making and action. These processes and their outcome act as an example of a pain experience for all involved and feedback into adapting or reinforcing beliefs, knowledge, and skills. The conceptual model also acknowledges the influence of the socio‐cultural context within which these experiences take place.

The findings illustrate several important considerations for those supporting or providing care for people with intellectual disability when they are in pain. There is a need for appropriate adaptation of communication methods and approaches to meet the individual needs of people with intellectual disability. This would also support the building of trusting relationships and may reduce some of the fears about contact with health services which were present within the data. Indeed, some of the reviewed studies described the role of non‐verbal pain appraisal when others could not rely on verbal information to assess pain. This requires the person with intellectual disability to feel safe within a consistent and reliable relationship with the person assessing the pain (Donovan 2002; Findlay et al. 2015). Being seen for a quick clinical appointment by an unknown person is the opposite of this. We believe that healthcare settings need to consider carefully how relationship building is prioritised in pain assessment and management for people with intellectual disability.

Pain assessment was highlighted as an area of difficulty within the findings, and there were indications that pain is not routinely explored (Drozd et al. 2020, 2021; Findlay et al. 2014). Directly enquiring about pain may facilitate open discussions about pain experiences and offer earlier opportunities to provide support and care when needed (Donovan 2002). Particularly in situations where pain is likely to occur, for example during known painful procedures, health professionals should be asking about pain and should remain attuned to potential pain cues at these times (Donavan 2022). Pain management strategies should be followed up and re‐assessed to establish whether they are effective for the individual, or whether alternative strategies need to be employed. Formal carers and health professionals should seek to become aware of their own beliefs about people with intellectual disability and pain, and to engage in appropriate learning and development where these are reflective of outdated or prejudiced assumptions.

The aforementioned considerations are in line with recommendations for healthcare provision for people with intellectual disability. For example, in England the ‘All our Health’ guidance has been published to help professionals understand and implement interventions to improve health and wellbeing, recognising the health inequalities that exist for this group: hospital passports and health actions plans are recommended to ensure that people with intellectual disability are well supported with their physical health and wellbeing, across their lifespan (Department of Health and Social Care 2025). Prevention and treatment of pain in vulnerable populations is increasingly seen as a global public health concern. In 2019, the IASP selected vulnerable populations for the focus for that year of campaigning, centring on adults with dementia, children, survivors of torture and people with psychiatric disorders, as well as people with intellectual disability (International Association for the Study of Pain 2019). The results outlined in the current review link with some similar experiences described by these other groups, and the issues around pain communication, assessment and treatment may also be applicable to them and the professionals who care for them.

Regarding further research, there remains a limited amount of qualitative data on this topic, and this area would benefit from further exploration. In relation to the conceptual model, further research examining the feedback loop would be beneficial in understanding how the different factors impact on pain experiences from the perspectives of people with intellectual disability. As highlighted in El‐Tallawy et al.'s (2023) review, there is increasing work on development of pain assessment tools and improving pain practices for people with intellectual disability. Their practical tips for the management of pain in intellectual disability are a helpful resource for professionals and families (El‐Tallawy et al. 2023). However, there is limited research conducted **with** people with intellectual disability examining what may be helpful and unhelpful in the assessment and management of pain. Additionally, as the wider biopsychosocial impact of pain was recognised within the included studies in this systematic review, further research examining this with people with intellectual disability, including things that help to manage these implications, would also be beneficial.

Finally, this review has highlighted the potential existence of ‘pain myths’ related to people with intellectual disability, such as the belief that they may have a higher pain tolerance. These myths may be rooted in stigma, as people with intellectual disability often face significant discrimination. Persistent pain can exacerbate this discrimination, particularly in the absence of biomedical evidence (De Ruddere et al. 2014) thus putting people with intellectual disability in a position of double disadvantage due to the intersection of their physical health difficulties and intellectual disability. Given this, future research could explore these factors in more detail.

### 4.2 Limitations

The transferability of the findings and synthesis is a limitation of this review. This is in relation both to the small number of studies meeting the eligibility criteria, the small sample sizes within each study owing to qualitative methodologies, and the synthesis representing the reviewer's understanding and conceptualisation of the data. Additionally, the studies included in the review did not represent a culturally diverse sample and so the meta‐ethnography does not offer insight into the phenomenon of pain within different cultures.

There were limitations within the included studies. Of particular importance was the lack of consideration for the researchers' own perspectives within the studies and analysis. This presents potential issues with the synthesis of the data and the ability to offer any further theoretical insights, as the reviewer was not able to consider the context of the data within the context of the original researcher's perspective. This may have changed how data was translated and synthesised, as there would have been a greater understanding of how the researcher had initially analysed and understood the data.

An additional limitation relates to the triangulation processes. Although these were put in place for study inclusion and quality appraisal, it may have been beneficial to also include this during the data translation and synthesis process. This could have offered alternative perspectives and further reduced bias regarding themes drawn out of the data, the way in which data was grouped, and where similarities and differences were observed within and between studies. Although this is a potential limitation, the first author outlined her epistemological position and views this review and synthesis as being representative of one way of understanding the available data.

Finally, it is possible that the inclusion criteria were a limitation. Only research which specifically explored the topic of pain, or within which the topic of pain was explicitly discussed, was included in the review. It may be advantageous for future reviews to employ more lenient criteria to encapsulate broader definitions and understandings of pain.

## 5 Conclusions

This review highlights some of the experiences and understandings regarding the phenomenon of pain within the adult LD population. Synthesising data from the included studies offered some further exploration of how pain is understood and approached by different individuals involved, including people with intellectual disability, their parents, carers, and health professionals. As there are only a small number of qualitative studies examining this topic, there is limited depth to the available data which contributed to difficulties in establishing further conceptual understandings of this phenomenon. In addition, research is focused on a UK population. However, we were able to propose a tentative model of factors which may be involved in a continual learning process about the meaning and experience of pain for people with intellectual disability. Further research is needed to fully understand how these factors impact on how pain is understood and experienced by people with intellectual disability, including identifying actions and decisions which are seen as harmful or helpful. There are clear individual differences in the experience of pain, and ways that it is approached, both by people with intellectual disability and those supporting people with intellectual disability; and this should inform the focus of further research. While developing tools and measures to support communication and health professionals' assessment of pain is important, as has been explored in previous studies, recognition must be given to wider factors which likely impact on whether, how, and when such tools would be used, and whether they are viewed as helpful by people with intellectual disability themselves.

## Conflicts of Interest

The authors declare no conflicts of interest.

## Data Availability

Research data are not shared.

## References

[jar70093-bib-0001] Breau, L. M. , C. S. Camfield , P. J. McGrath , and G. A. Finley . 2003. “The Incidence of Pain in Children With Severe Cognitive Impairments.” Archives of Pediatrics & Adolescent Medicine 157, no. 12: 1219–1226. 10.1001/archpedi.157.12.1219. 14662579

[jar70093-bib-0034] Campbell, R. , P. Pound , M. Morgan , et al. 2011. “Evaluating Meta‐Ethnography: Systematic Analysis and Synthesis of Qualitative Research.” Health Technology Assessment 15, no. 43. 10.3310/hta15430. 22176717

[jar70093-bib-0002] Chester, V. , and M. Henriksen . 2014. “Pain Experience and Management in a Forensic Intellectual Disability Service.” Advances in Mental Health and Intellectual Disabilities 8, no. 2: 120–127. 10.1108/AMHID-03-2013-0026.

[jar70093-bib-0003] Clarke, Z. J. , and A. R. Thompson . 2007. “Parents' Experiences of Pain and Discomfort in People With Learning Disabilities.” British Journal of Learning Disabilities 36: 84–90. 10.1111/j.1468-3156.2007.00467.x.

[jar70093-bib-0004] Critical Appraisal Skills Programme . 2018. “CASP Qualitative Checklist.” https://casp‐uk.net/images/checklist/documents/CASP‐Qualitative‐Studies‐Checklist/CASP‐Qualitative‐Checklist‐2018_fillable_form.pdf.

[jar70093-bib-0005] De Knegt, N. , and E. Scherder . 2011. “Pain in Adults With Intellectual Disabilities.” Pain 152: 971–974. 10.1016/j.pain.2010.11.001. 21112699

[jar70093-bib-0006] De Ruddere, L. , L. Goubert , M. A. L. Stevens , M. Deveugele , K. D. Craig , and G. Crombez . 2014. “Health Care Professionals' Reactions to Patient Pain: Impact of Knowledge About Medical Evidence and Psychosocial Influences.” Journal of Pain 15, no. 3: 262–270. 10.1016/j.jpain.2013.11.002. 24275317

[jar70093-bib-0007] Department of Health and Social Care . 2025. “Learning Disability – Applying All Our Health.” https://www.gov.uk/government/publications/learning‐disability‐applying‐all‐our‐health/learning‐disabilities‐applying‐all‐our‐health#:~:text=Compared%20to%20people%20without%20a,in%20people%20with%20learning%20disabilities.

[jar70093-bib-0008] Donovan, J. 2002. “Learning Disability Nurses' Experience of Being With Clients in Pain.” Issues and Innovations in Nursing Practice 38, no. 5: 458–466. 10.1046/j.1365-2648.2002.02207.x. 12028279

[jar70093-bib-0009] Drozd, M. , D. Chadwick , and R. Jester . 2020. “A Cross‐Case Comparison of the Trauma and Orthopaedic Hospital Experiences of Adults With Intellectual Disabilities Using Interpretative Phenomenological Analysis.” Nursing Open 8, no. 2: 858–869. 10.1002/nop2.693. 33570307 PMC7877147

[jar70093-bib-0010] Drozd, M. , D. Chadwick , and R. Jester . 2021. “The Voices of People With an Intellectual Disability and a Carer About Orthopaedic and Trauma Hospital Care in the UK: An Interpretative Phenomenological Study.” International Journal of Orthopaedic and Trauma Nursing 42, no. 2021: 1–11. 10.1016/j.ijotn.2020.100831. 33563567

[jar70093-bib-0011] El‐Tallawy, S. N. , R. S. Ahmed , and M. S. Nagiub . 2023. “Pain Management in the Most Vulnerable Intellectual Disability: A Review.” Pain and Therapy 12: 939–961. 10.1007/s40122-023-00526-w. 37284926 PMC10290021

[jar70093-bib-0033] Elo, S. , and H. Kyngäs , 2008. “The Qualitative Content Analysis Process.” Journal of Advanced Nursing 62, no. 1: 107–115. Portico. 10.1111/j.1365-2648.2007.04569.x. 18352969

[jar70093-bib-0012] Findlay, L. , A. C. d. C. Williams , and K. Scior . 2014. “Exploring Experiences and Understandings of Pain in Adults With Intellectual Disabilities.” Journal of Intellectual Disability Research 58, no. 4: 358–367. 10.1111/jir.12020. 23356659

[jar70093-bib-0013] Findlay, L. , A. C. d. C. Williams , and K. Scior . 2015. “Caregiver Experiences of Supporting Adults With Intellectual Disabilities in Pain.” Journal of Applied Research in Intellectual Disabilities 28, no. 2: 111–120. 10.1111/jar.12109. 24909927

[jar70093-bib-0014] Gabre, P. , and K. Sjoquist . 2002. “Experience and Assessment of Pain in Individuals With Cognitive Impairments.” Special Care in Dentistry 22, no. 5: 174–180. 10.1111/j.1754-4505.2002.tb00267.x. 12580355

[jar70093-bib-0015] Goodall, M. , K. Irving , and M. Nevin . 2023. “The Recognition, Assessment and Perceptions of Total Pain in People With Profound Intellectual Disabilities: A Mixed Methods Systematic Review.” Journal of Applied Research in Intellectual Disabilities 36, no. 5: 940–950. 10.1111/jar.13132. 37365750

[jar70093-bib-0016] Hadi, M. A. , G. A. McHugh , and S. J. Closs . 2019. “Impact of Chronic Pain on Patients' Quality of Life: A Comparative Mixed‐Methods Study.” Journal of Patient Experience 6, no. 2: 133–141. 10.1177/2374373518786013. 31218259 PMC6558939

[jar70093-bib-0017] International Association for the Study of Pain . 2019. “Pain in the Most Vulnerable.” https://www.iasp‐pain.org/advocacy/global‐year/pain‐in‐the‐most‐vulnerable/.

[jar70093-bib-0018] International Association for the Study of Pain . 2020. “IASP Announces Revised Definition of Pain.” https://www.iasp‐pain.org/publications/iasp‐news/iasp‐announces‐revised‐definition‐of‐pain/.

[jar70093-bib-0019] Long, H. A. , D. P. French , and J. M. Brooks . 2020. “Optimising the Value of the Critical Appraisal Skills Programme (CASP) Tool for Quality Appraisal in Qualitative Evidence Synthesis.” Research Methods in Medicine & Health Sciences 1, no. 1: 31–42. 10.1177/2632084320947559.

[jar70093-bib-0020] McGuire, B. E. , and S. Kennedy . 2013. “Pain in People With an Intellectual Disability.” Current Opinion in Psychiatry 26, no. 3: 270–275. 10.1097/yco.0b013e32835fd74c. 23508000

[jar70093-bib-0021] Millard, S. K. , and N. C. De Knegt . 2019. “Cancer Pain in People With Intellectual Disabilities: Systematic Review and Survey of Health Care Professionals.” Journal of Pain and Symptom Management 58, no. 6: 1081–1099. 10.1016/j.jpainsymman.2019.07.013. 31326504

[jar70093-bib-0022] Noblit, G. W. , and R. D. Hare . 1988. Meta‐Ethnography: Synthesising Qualitative Studies. Sage Publications.

[jar70093-bib-0023] Nocon, A. , L. Sayce , and Z. Nadirshaw . 2008. “Health Inequalities Experienced by People With Learning Disabilities: Problems and Possibilities in Primary Care.” Tizard Learning Disability Review 13, no. 1: 28–36. 10.1108/13595474200800005.

[jar70093-bib-0024] Olmos‐Vega, F. M. , R. E. Stalmeijer , L. Varpio , and R. Kahlke . 2022. “A Practical Guide to Reflexivity in Qualitative Research: AMEE Guide No. 149.” Medical Teacher 45, no. 3: 241–251. 10.1080/0142159X.2022.2057287. 35389310

[jar70093-bib-0025] Page, M. J. , D. Moher , P. M. Bossuyt , et al. 2021. “The PRISMA 2020 Statement: An Updated Guideline for Reporting Systematic Reviews.” BMJ 372, no. 71: 1–9. 10.1136/bmj.n71. PMC8005924 33782057

[jar70093-bib-0031] Robertson, J. , S. Baines , E. Emerson , and C. Hatton . 2017. “Constipation Management in People With Intellectual Disability: A Systematic Review.” Journal of Applied Research in Intellectual Disabilities 31, no. 5: 709–724. Portico. 10.1111/jar.12426. 29168259

[jar70093-bib-0032] Smith, J. A. , P. Flowers , and M. Larkin . 2009. Interpretative Phenomenological Analysis: Theory, Method and Research. Sage.

[jar70093-bib-0027] Symons, F. J. , S. K. Shinde , and E. Gilles . 2008. “Perspectives on Pain and Intellectual Disability.” Journal of Intellectual Disability Research 52, no. 4: 275–286. 10.1111/j.1365-2788.2007.01037.x. 18205754

[jar70093-bib-0028] Toye, F. , K. Seers , N. Allcock , M. Briggs , E. Carr , and K. Barker . 2014. “Meta‐Ethnography 25 Years on: Challenges and Insights for Synthesising a Large Number of Qualitative Studies.” BMC Medical Research Methodology 14, no. 80: 1–14. 10.1186/1471-2288-14-80. 24951054 PMC4127190

[jar70093-bib-0029] Walsh, M. , T. G. Morrison , and B. W. McGuire . 2011. “Chronic Pain in Adults With an Intellectual Disability: Prevalence, Impact, and Health Service Use Based on Caregiver Report.” Pain 152, no. 9: 1951–1957. 10.1016/j.pain.2011.02.031. 21497999

[jar70093-bib-0030] Yeganeh, H. , Z. Su , and E. Chrysostome . 2004. “A Critical Review of Epistemological and Methodological Issues in Cross‐Cultural Research.” Journal of Comparative International Management 7, no. 2: 66–86.

